# The impact of genetic polymorphisms in xenobiotic-metabolizing enzymes and the risk of oral cancer: a critical review

**DOI:** 10.1007/s12032-026-03428-x

**Published:** 2026-10-08

**Authors:** Gabriel Carvalhal de Aguiar, Daniel Vitor de Souza, Barbara dos Anjos Rosário, Lorrany da Silva Avanci, Antonio Silva, Rogerio Aparecido Dedivitis, Daniel Araki Ribeiro

**Affiliations:** 1 Department of Biosciences, Institute of Health and Society, Federal University of São Paulo, UNIFESP, Rua Silva Jardim, 136, Room 332, Vila Mathias, Santos, SP 11050-020 Brazil; 2 Department of Head and Neck Surgery, Faculty of Medicine, Sao Paulo University, (USP), São Paulo, SP Brazil

**Keywords:** Oral squamous cell carcinoma, Cytochrome P450 polymorphisms, Glutathione S-transferase polymorphisms, Gene–environment interaction

## Abstract

Oral squamous cell carcinoma (OSCC) and oral potentially malignant disorders (OPMDs) affect many individuals, where exposure to xenobiotics such as tobacco, alcohol, and betel quid is highly prevalent. Inter-individual variability plays a key role regarding genetic polymorphisms in xenobiotic‑metabolizing enzymes. In this study, we searched PubMed, Scopus and Web of Science (up to April 2026) for observational studies and meta‑analyses evaluating CYP1A1, CYP1B1, CYP2E1, GSTM1, GSTT1, GSTP1 and GSTM3 polymorphisms in OSCC and OPMDs. Twenty-one studies met the inclusion criteria. Data on exposures, genotypes and risk estimates were extracted and synthesized qualitatively. Most studies showed that GSTM1 and/or GSTT1 null genotypes are associated with increased risk of OSCC, particularly in Asian cohorts with heavy tobacco and/or betel quid exposure. CYP1A1 and CYP2E1 variants exhibited more heterogeneous results, but several studies indicated elevated risk in smokers, drinkers and betel quid users. Stronger effects consistently appeared in gene–environment interaction models. Polymorphisms in Phase I and Phase II metabolizing enzymes, especially GSTM1/GSTT1 null and selected CYP1A1/CYP2E1 variants, modulate susceptibility to OSCC in exposed individuals, supporting their potential use in risk stratification and targeted prevention strategies.

**Supplementary Information:**

The online version contains supplementary material available at https://doi.org/10.1007/s12032-026-03428-x.

## Introduction

Oral cancer is one of the most devastating malignancies affecting the head and neck region, combining high incidence with profound functional, aesthetic, and psychosocial consequences for affected individuals [1]. According to recent global cancer statistics, cancers of the lip and oral cavity together account for nearly 400,000 new cases and over 180,000 deaths annually, with a marked concentration of this burden in low‑ and middle‑income countries [2]. South and Southeast Asia, in particular, exhibit some of the highest incidence rates worldwide, closely mirroring the regional prevalence of tobacco use, alcohol consumption, and betel quid or areca nut chewing [3]. Within this epidemiological landscape, oral squamous cell carcinoma (OSCC) represents the predominant histological subtype and frequently develops on a background of oral potentially malignant disorders (OPMDs) such as leukoplakia and oral submucous fibrosis (OSF), which themselves confer a substantially increased risk of malignant transformation [4, 5].

The etiological paradigm of oral carcinogenesis is classically anchored in exposure to well‑established environmental risk factors. The most important xenobiotic for oral cancer risk is tobacco smoke that contains a complex mixture of polycyclic aromatic hydrocarbons, nitrosamines, aldehydes and other electrophilic compounds that can induce DNA damage in oral epithelial cells [3]. Alcohol also presents an important chemical agent able to act not only as a solvent that facilitates mucosal penetration of carcinogens, but also as a substrate for the generation of acetaldehyde, a recognized mutagenic metabolite [3]. Additionally, other agents such as betel quid and areca nut have been classified by the International Agency for Research on Cancer (IARC) as Group 1 carcinogens, with robust epidemiological evidence linking their chronic use to OPMDs and cancers of the oral cavity and pharynx [6]. Meta-analyses indicate that habitual betel quid chewers experience a relative risk of oral cancer five to eight times higher than non-users, and this risk is even higher when tobacco and alcohol are also consumed [7]. Nevertheless, only a fraction of exposed individuals progress to OPMDs or OSCC, strongly suggesting that intrinsic host factors critically modulate the carcinogenic impact of these exogenous agents [8, 9].

A major component of this host modulation resides in genetic polymorphisms of xenobiotic‑metabolizing enzymes, particularly those involved in the activation and detoxification of tobacco‑ and areca‑derived pro-carcinogens. Phase I cytochrome P450 (CYP) enzymes such as CYP1A1, CYP1B1 and CYP2E1 catalyze oxidative biotransformation reactions that can convert relatively inert compounds into highly reactive electrophilic intermediates capable of forming bulky DNA adducts and inducing DNA strand breaks [10]. In contrast, Phase II glutathione S‑transferases (GSTs), including GSTM1, GSTT1, GSTP1 and GSTM3, conjugate these intermediates with glutathione, thereby increasing their water solubility and facilitating their excretion [10]. Functional polymorphisms in these genes, such as null deletions of GSTM1 and GSTT1, the GSTP1 Ile105Val and GSTM3 A/B variants, and high‑activity alleles of CYP1A1 and CYP2E1, can modulate the biochemical balance toward the accumulation rather than the clearance of DNA‑reactive species, amplifying the genotoxic burden in oral tissues chronically exposed to chemical carcinogens [11, 12].

A substantial body of genetic epidemiology has explored these hypotheses in populations with high exposure to some xenobiotics such as tobacco, alcohol and betel quid. Case–control studies from India, Japan, Taiwan and China have repeatedly linked GSTM1 null and GSTT1 null genotypes to increased risks of oral leukoplakia, OSCC and head and neck squamous cell carcinoma (HNSCC), often reporting odds ratios above two‑fold under conditions of intense tobacco smoking or betel quid chewing [13, 14]. Similar evidence has emerged for coding variants in GSTP1 and GSTM3, which have been associated with increased risk of OSCC and OPMDs in several Indian cohorts [15, 16]. In parallel, multiple investigations have shown that CYP1A1 Ile462Val and CYP2E1 c2 alleles are associated with elevated oral cancer risk, particularly among heavy smokers and drinkers or in individuals chronically exposed to areca‑related nitrosamines [17]. Importantly, some of these studies do not merely report main genetic effects; they emphasize gene–environment interactions, such as the stronger impact of CYP2E1 variants in non‑betel chewers or the selective effect of GSTA1 variants among male smokers [18].

However, the literature in this field is still controversial. This is due to the fact that some investigations have failed to confirm associations between GSTM1 or GSTT1 null genotypes and oral cancer, or have reported null or even protective effects for specific CYP variants in certain ethnic groups or exposure strata [19, 20]. Meta‑analyses focused on individual polymorphisms, such as CYP1A1 Ile462Val or GSTM1 null, generally support a modest but significant increase in oral cancer risk, especially in Asian populations, but frequently pool heterogeneous tumor sites, exposure patterns and clinical endpoints, and rarely incorporate OPMDs alongside OSCC in a coherent biological rationale for oral carcinogenesis [21–23]. Moreover, most previous reviews have examined Phase I and Phase II pathways in isolation, without critically addressing their combined effect or mapping them onto clearly defined exposure profiles involving tobacco, alcohol and betel quid [24].

In light of these considerations, there is a clear need for a comprehensive and methodologically rigorous synthesis that integrates Phase I (CYP1A1, CYP1B1, CYP2E1) and Phase II (GSTM1, GSTT1, GSTP1, GSTM3) polymorphisms, considers both OSCC and OPMDs as multi-step process of oral carcinogenesis, and explicitly evaluates gene–environment interactions in populations heavily exposed to recognized risk factors [24]. The present review was therefore designed to critically evaluate the association between polymorphisms in key Phase I and Phase II metabolizing enzymes and susceptibility to OSCC and OPMDs, with particular emphasis on gene–environment interactions involving tobacco, alcohol and betel quid when applicable. Through stratified analyses by enzyme class, clinical phenotype and exposure profile, this work aims to delineate biologically coherent patterns of risk and to identify genetic–environmental conditions that may be clinically useful for risk stratification and targeted prevention in high‑burden populations.

## Material and methods

### Scope and literature selection

To construct this critical review, we focused on identifying observational case-control studies, cohort studies, and meta-analyses that assessed genetic susceptibility in human populations. The selection prioritized articles that investigated polymorphisms in Phase I and Phase II metabolizing enzymes associated with oral oncogenesis. We selected English language publications that provided clear genotypic or allelic frequencies, validated molecular genotyping protocols, and sufficient data to discuss risk estimates. Editorials, case reports, or studies lacking proper control groups were not prioritized for this narrative synthesis, as our goal was to critically evaluate robust empirical evidence regarding gene-environment interactions.

Because this is a critical narrative review, the included literature was qualitatively appraised for its relevance to the biological pathways of interest. We focused on the strength of the reported gene-environment interactions rather than applying a formal quantitative risk of bias scoring system, allowing for a comprehensive discussion of the metabolic and oncogenic mechanisms.

### Search strategy

A comprehensive literature search was conducted in April 2026 using electronic databases such as PubMed, Scopus, and Web of Science. The search strategy involved the combination of key terms and Medical Subject Headings (MeSH) using Boolean operators, as follows: ("Oral cancer" OR "Oral squamous cell carcinoma" OR OSCC OR "Oral potentially malignant disorders" OR Leukoplakia OR "Oral submucous fibrosis") AND (Polymorphism OR "Single nucleotide polymorphism" OR SNP OR Genotype) AND ("Cytochrome P450" OR CYP1A1 OR CYP2E1 OR "Glutathione S-transferase" OR GSTM1 OR GSTT1 OR GSTP1 OR "Metabolizing enzymes").

To ensure a thorough review, manual reference checking was performed on articles selected during the initial screening. No restrictions were placed on the publication date.

### Study selection process

Five independent reviewers (GCA, DVS, BAR, LSA and DAR) conducted the initial screening of titles and abstracts to crosscheck the data. The full text of each potentially eligible article was retrieved for further evaluation. Disagreements among reviewers were resolved through discussion and consensus.

To address the inherent biological differences among the metabolic pathways, the included studies were strictly stratified into two distinct subgroups for data synthesis and discussion: (1) Phase I bioactivation enzymes (CYP family) and (2) Phase II detoxification enzymes (GST family). The complete search strategy and the selection flow are detailed in Table 1.

**Table 1:** Search strategy

Electronic databases used	Search strategy (April, 2026)
PubMed https://www.ncbi.nlm.nih.gov/pubmed/ Scopus https://www.scopus.com Web of Science https://www.webofscience.com/wos/alldb/	(“Oral cancer” OR “Oral squamous cell carcinoma” OR OSCC OR “Oral potentially malignant disorders” OR Leukoplakia OR “Oral submucous fibrosis”) AND (Polymorphism OR “Single nucleotide polymorphism” OR SNP OR Genotype) AND (“Cytochrome P450” OR CYP1A1 OR CYP2E1 OR “Glutathione S-transferase” OR GSTM1 OR GSTT1 OR GSTP1 OR “Metabolizing enzymes”)

## Results

### Study selection

The initial online search across the selected electronic databases yielded a total of 21 studies included in this systematic review.

To accommodate the biological heterogeneity of the metabolic pathways evaluated and to provide a structured data synthesis, the final sample of included studies was stratified into two predefined biological subgroups: Phase I bioactivation enzymes (primarily the Cytochrome P450 family, including CYP1A1, CYP1B1, and CYP2E1) and Phase II detoxification enzymes (primarily the Glutathione S-transferase family, including GSTM1, GSTT1, GSTP1, and GSTM3).

### General characteristics of the included studies

Table 2 summarizes the primary demographic and methodological characteristics of the 21 included studies. The selected literature spans a broad timeframe from 1997 to 2024, demonstrating a consistent and global scientific interest in the genetic susceptibility to oral cancer. Geographically, there is a pronounced concentration of research in the Asian continent, which strongly aligns with the high regional incidence of the disease and the genetic predisposition of these populations. India contributed the most substantial portion of the data with twelve studies, followed by significant cohorts from China, Japan, and Taiwan. Western populations were also represented, albeit to a lesser extent, through cohorts from Germany and Canada.

**Table 2:** Main demographic and methodological characteristics of the included studies

Author and year	Country	Ethnicity	Study design	Clinical condition	Sample size (Cases/Controls)
ThekkePurakkal et al. [25]	Canada	Caucasians	Case–control	HNSCC	389/429
Maurya et al. [17]	India	Indo-European	Case–control	HNSCC	750/750
Zhuo et al. [21]	China	Multiple	Meta-analysis	OSCC	1515/2233
Balaji et al. [19]	India	South Asian	Case–control	OSCC	157/132
Katoh et al. [12]	Japan	Japanese	Case–control	OSCC	92/147
Hung et al. [9]	Taiwan	Asian	Case–control	OSCC	41/123
Bartake et al. [26]	India	South Asian	Case–control	OED and OSCC	44/44
Singh et al. [27]	India	Indian (Gujarat)	Case–control	OSCC	121/121
Katiyar et al. [13]	India	Indo-European	Case–control	HNSCC	1250/1250
Li et al. [22]	China	Chinese	Meta-analysis	OSCC	1306/1484
Rajesh et al. [16]	India	Indian	Case–control	OSCC	90/180
Krüger et al. [20]	Germany	Caucasian	Case–control	OSCC	100/93
Singh et al. [28]	India	Indian (Gujarati)	Case–control	OSCC	122/127
Koch et al. [29]	Germany	Caucasian	Case-series	OSCC	28/0
Chen et al. [30]	Taiwan	Taiwanese	Case–control	OSCC	164/274
Bathi et al. [31]	India	Indian	Case–control	Oral cancer, Leukoplakia, OSF	90/60
Chatterjee et al. [32]	India	Indian (Tribal/Nontribal)	Case–control	Leukoplakia	52/217
Datta et al. [15]	India	Indian (Bengalee)	Case–control	OSCC and Leukoplakia	310/389
Komiya et al. [18]	Japan	Japanese	Case–control	OSCC	97/457
Sikdar et al. [14]	India	Indian (Bengalee)	Case–control	OSCC and Leukoplakia	256/259
Buch et al. [11]	India	Indian	Case–control	OSCC	297/450

HNSCC = Head and neck squamous cell carcinoma; OSCC = Oral squamous cell carcinoma, OED = Oral epithelial dysplasia; OSF = Oral submucous fibrosis

The clinical conditions investigated across the studies primarily encompassed Oral Squamous Cell Carcinoma (OSCC) and broader Head and Neck Squamous Cell Carcinomas (HNSCC). Additionally, several studies meticulously evaluated potentially malignant disorders, such as Oral Leukoplakia, Oral Epithelial Dysplasia (OED), and Oral Submucous Fibrosis (OSF), providing a comprehensive view of the oncological spectrum. Consequently, throughout our narrative synthesis, we made a deliberate effort to distinguish findings specific to isolated OSCC from broader HNSCC cohorts, as well as separating distinct OPMD entities like Leukoplakia and OSF.

Regarding the methodological design, the majority of the sample consists of eighteen case–control studies, alongside one case-series and two robust meta-analyses [21, 22]. The sample sizes exhibited significant variation, ranging from smaller primary investigations, such as Koch et al. [29] with 28 cases, to extensive epidemiological meta-analyses that pooled thousands of individuals, significantly increasing the statistical power to address genetic controversies in the literature. It is important to note that while we included two meta-analyses to highlight large-scale pooled estimates, care was taken in our narrative synthesis to discuss these separately from the primary case–control cohorts to avoid double-counting of the underlying patient populations.

### Distribution of risk factors and demographic profiles

Table 3 details the sociodemographic variables and the prevalence of established risk factors among the evaluated cohorts. A pronounced male predominance was observed across the majority of the studies. Notably, the investigations conducted by Maurya et al. [17], Hung et al. [9], and Katiyar et al. [13] utilized cohorts composed entirely of male participants. The age of the participants varied widely, generally reflecting the typical onset of oral cancer in the fifth and sixth decades of life.

**Table 3:** Risk factors and habits

Author and year	Age (Cases/Controls)	Gender (Cases/Control)	Tobacco Smoking	Alcohol consumption	Betel quid/smokeless tabacco
ThekkePurakkal et al. [25]	61/61 years	291 M, 98 F/298 M, 131 F	Yes (Evaluated in pack years: nonsmokers, moderate, heavy)	Yes (Ethanol frequency evaluated in ml/day)	N/A
Maurya et al. [17]	53 ± 9/54 ± 6 years	750 M, 0 F/750 M, 0 F (Only male sample)	Yes (67% cases/29% controls)	Yes (42% cases/31% controls)	Yes (Tobacco chewing: 53% cases/32% controls)
Zhuo et al. [21]	N/A	N/A	N/A (Meta analysis did not provide isolated statistics)	N/A	N/A
Balaji et al. [19]	55.1/53.1 years	54.8% M/34.8% M	Evaluated and matched	Evaluated and matched	N/A
Katoh et al. [12]	62.4/69.9 years	60.9% M/61.9% M	Yes (63% cases/34% controls)	Yes (56.7% cases/53.1% controls)	N/A
Hung et al. [9]	54.1/51.7 years	Only male sample (100% M)	Yes (90.2% cases/65.8% controls)	Yes (65.9% cases/40.7% controls)	Yes (73.2% cases/12.2% controls)
Bartake et al. [26]	54.6 (OSCC) and 49.6 (OED)/43.2 (CWT) and 37.6 (CWOT)	OSCC (72.7% M) and OED (86.4% M)/CWT (63.6% M) and CWOT (36.4% M)	Yes (All cases and 50% of controls used tobacco)	Not evaluated as grouping variable	N/A
Singh et al. [27]	42.6/40.2 years	81.2% M/92.3% M	Yes (Included in general tobacco use: 85.1% cases/47.9% controls)	Yes (Considered as risk factor, but frequency not detailed)	Yes (Mentioned gutkha and pan masala)
Katiyar et al. [13]	44.47/51.55 years	100% M/100% M	Yes (Smokers: 67.6% cases/29.3% controls)	Yes (35.1% cases/25.9% controls)	Yes (Tobacco chewers: 52% cases/32% controls)
Li et al. [22]	N/A	N/A	N/A	N/A	N/A
Rajesh et al. [16]	54.2 ± 12.03/53.7 ± 12.5 years	20 M, 70 F/43 M, 137F	Yes (Evaluated as overall tobacco consumption)	Yes (Only 5 cases reported alcohol use)	Yes (100% of cases used betel quid or chewable tobacco)
Krüger et al. [20]	Not informed	Not informed	Yes (Smokers vs Nonsmokers analyzed)	Not evaluated as primary variable	N/A
Singh et al. [28]	44.4 ± 1.0/32.2 ± 0.72 years	101 M, 21 F/116 M, 11F	Yes (85.2% cases/48.8% controls)	Evaluated as mixed habits (17.2% of cases)	Yes (Exclusive chewers: 54.9% cases/41.7% controls)
Koch et al. [29]	67/Not informed years	21 M, 7 F/N/A	Yes (Mentioned as general risk factor, no frequencies provided)	Yes (Mentioned as general risk factor)	N/A
Chen et al. [30]	53.35 ± 11.94/55.87 ± 17.50 years	Matched (frequencies not detailed in text)	Yes (Adjusted as confounding factor)	Yes (Adjusted as confounding factor)	Yes (Adjusted as confounding factor)
Bathi et al. [31]	21 to 75 years range	Matched (frequencies not detailed)	Yes (Control group divided into 30 smokers and 30 nonsmokers)	N/A	Yes (Cases had smoking, chewing or mixed habits)
Chatterjee et al. [32]	Mean 28.88 years for target population	Overall population: 68 M, 149F	Yes (Evaluated by shots/day)	N/A	Yes (Mentioned raw tobacco and betel nut usage)
Datta et al. [15]	55 ± 11/49 ± 12 years	198 M, 112 F/302 M, 87 F (For Cancer vs Control)	Yes (17% Cases/37% Controls)	Evaluated but excluded due to low frequency	Yes (57% Cases/44% Controls)
Komiya et al. [18]	62.8/67.2 years	62 M, 35 F/284 M, 173F	Yes (Adjusted as confounding factor)	Not collected for controls	N/A
Sikdar et al. [14]	55 ± 11.6/53 ± 11.0 years	168 M, 88 F/183 M, 76 F (For Cancer vs Control)	Yes (Evaluated as light/heavy pack years)	Excluded from analysis (low frequency)	Yes (Evaluated as light/heavy chewing years)
Buch et al. [11]	52.2 ± 0.69/42.9 ± 0.53 years	Not explicitly detailed in tables	Yes (Bidi and cigarette)	N/A	Yes (Chewing tobacco with or without betel quid)

HNSCC: Head and neck squamous cell carcinoma; OSCC: Oral squamous cell carcinoma; OED: Oral epithelial dysplasia; OSF: Oral submucous fibrosis; M: Male; F: Female; N/A: Not applicable or not available

Regarding environmental exposures, tobacco smoking and alcohol consumption were consistently evaluated as primary risk factors or confounding variables. Smoking prevalence was alarmingly high among the oncological patients; for instance, Hung et al. [9] and Singh et al. [28] reported tobacco use in 90.2% and 85.2% of their cases, respectively. Alcohol consumption mirrored this trend, acting as a synergistic risk factor in several populations.

Furthermore, the consumption of smokeless tobacco and betel quid emerged as a critical variable, particularly within the Asian cohorts. The data demonstrates a massive prevalence of this habit, with Rajesh et al. [16] reporting that 100% of their cases used chewable tobacco or betel quid. Similarly, Hung et al. [9] highlighted this exposure in 73.2% of the cases compared to only 12.2% in the control group, solidifying its role as a major catalyst for malignant transformation in these specific geographic regions.

### Genotyping methodology and investigated polymorphisms

Table 4 details the DNA sources and molecular techniques utilized to assess genetic variants. Peripheral blood (whole blood or leukocytes) was the most common source for genomic DNA extraction, ensuring high-quality genetic material, while some studies opted for non-invasive methods using oral exfoliated cells or brush biopsies. Polymerase Chain Reaction (PCR) based techniques were universally adopted. Specifically, PCR-Restriction Fragment Length Polymorphism (PCR–RFLP) and Multiplex PCR were the most frequently applied methods to determine the genotypes.

**Table 4:** Molecular methodologies and specific genetic polymorphisms evaluated

Author and year	Source of DNA	Genotyping method	Evaluated genes	Polymorphism/SNP
ThekkePurakkal et al. [25]	Oral exfoliated cells (buccal mucosa)	Real Time PCR (TaqMan)	CYP1A1, CYP2A6, CYP2E1, GSTP1, CYP2D6, GSTM1	CYP1A1*2A, CYP1A1*2C, CYP2A6*2, CYP2E1c2, GSTP1 105Val, null CYP2D6, GSTM1 null
Maurya et al. [17]	Whole blood	Multiplex PCR (GSTM1) and PCR RFLP (CYPs)	CYP1A1, CYP1B1, CYP2E1, GSTM1	CYP1A1*2A (Msp1), CYP1A1*2C (Ile/Val), CYP1B1*2, CYP1B1*3, CYP2E1*5B (RsaI), CYP2E1*6 (DraI), GSTM1 (null)
Zhuo et al. [21]	N/A	Various (PCR RFLP, AS PCR, PCR SSCP in primary studies)	CYP1A1	Ile462Val (exon7)/rs1048943
Balaji et al. [19]	Peripheral blood	TaqMan Fast Real Time PCR	CYP2E1	rs6413419 (V1791), rs3813867 (PstI), rs2031920 (RsaI)
Katoh et al. [12]	Peripheral leukocytes	Multiplex PCR (GSTs) and PCR RFLP (CYPs)	CYP1A1, CYP2E1, GSTM1, GSTT1, ALDH2	CYP1A1 (Ile462Val), CYP2E1 (RsaI c1/c2), GSTM1 (null), GSTT1 (null)
Hung et al. [9]	Peripheral white blood cells (WBCs)	PCR RFLP (CYP2E1) and PCR (GSTs)	CYP2E1, GSTM1, GSTT1	CYP2E1 (RsaI c1/c2), GSTM1 (null), GSTT1 (null)
Bartake et al. [26]	Whole blood (EDTA)	TaqMan RT PCR	CYP1B1	Leu432Val/L432V (rs1056836)
Singh et al. [27]	Peripheral blood lymphocytes	PCR RFLP and PCR SSCP	CYP1A1, CYP2E1, GSTM1, GSTT1, p53, MDM2	CYP1A1*2A (rs1048943), CYP1A1*2C (rs4646903), CYP2E1*5B, CYP2E1*6 (rs6413432), GSTM1 (null), GSTT1 (null), p53 (rs17878362, rs1042522, rs1625895), MDM2 (rs2279744)
Katiyar et al. [13]	Whole blood	Multiplex PCR (GSTM1, GSTT1) and PCR RFLP (GSTP1)	GSTM1, GSTT1, GSTP1	GSTM1 (null), GSTT1 (null), GSTP1 (Ile105Val)
Li et al. [22]	N/A	Meta analysis	GSTM1, GSTT1	Null genotypes
Rajesh et al. [16]	Tumor specimens (Cases) and Peripheral blood (Controls)	PCR RFLP	GSTP1	c.341C>T (rs1138272)
Krüger et al. [20]	Lymphocytes from venous blood (Cases) and Brush biopsy (Controls)	Real time PCR (TaqMan)	GSTM1, GSTT1	Null genotypes (Deletion)
Singh et al. [28]	Peripheral lymphocytes	PCR RFLP (CYP1A1*2A), PCR SSCP (CYP1A1*2C), and Multiplex PCR (GSTs)	CYP1A1, GSTM1, GSTT1	CYP1A1*2A (MspI), CYP1A1*2C (Ile/Val), GSTM1 (null), GSTT1 (null)
Koch et al. [29]	Peripheral blood (for DNA) and Brush biopsy (for mRNA)	PCR (for DNA) and Real Time qPCR (for mRNA)	GSTM1	Null genotype (homozygous deletion)
Chen et al. [30]	Whole blood	PCR RFLP	GSTP1, GSTA1, GSTT1, GSTM1	GSTP1 (Ile105Val/A>G), GSTA1 (C>T at 69), GSTT1 (null), GSTM1 (null)
Bathi et al. [31]	Peripheral blood leukocytes	PCR	GSTM1, GSTT1	Null genotypes
Chatterjee et al. [32]	Peripheral blood leukocytes	PCR RFLP (CYP1A1) and Multiplex PCR (GSTs)	CYP1A1, GSTM1, GSTT1	CYP1A1 (MspI: m1/m2), GSTM1 (null), GSTT1 (null)
Datta et al. [15]	Whole blood (and affected tissues)	PCR RFLP	Mitochondrial loci, GSTP1, GSTM1, GSTM3	mt A12308G, A11467G, G10398A, C10400T; GSTs (various)
Komiya et al. [18]	Peripheral leukocytes	PCR RFLP	GSTA1	*A/*B (*A: 567 T, 69 C, 52G, *B: 567G, 69 T, 52 A)
Sikdar et al. [14]	Whole blood	Multiplex PCR, PCR, and PCR RFLP	GSTM1, GSTT1, GSTM3, GSTP1	GSTM1 (null), GSTT1 (null), GSTM3 (3 bp deletion A/B), GSTP1 (Ile105Val, Ala114Val)
Buch et al. [11]	White blood cells	PCR and PCR RFLP	GSTM1, GSTM3, GSTT1	GSTM1 (null), GSTM3 (A/B), GSTT1 (null)

PCR: Polymerase chain reaction; PCR RFLP: Polymerase chain reaction restriction fragment length polymorphism; PCR SSCP: Polymerase chain reaction single strand conformation polymorphism; AS PCR: Allele specific polymerase chain reaction; RT PCR: Real time polymerase chain reaction; CYP: Cytochrome P450; GST: Glutathione S transferase; ALDH2: Aldehyde dehydrogenase 2; MDM2: Mouse double minute 2 homolog; SNP: Single nucleotide polymorphism

The studies predominantly investigated genes involved in carcinogen metabolism, highlighting the critical role of these enzymes in the neutralization of electrophilic compounds and tobacco-derived procarcinogens. The most frequently evaluated Phase I enzymes were CYP1A1 (specifically the *2A and *2C variants) and CYP2E1. For Phase II detoxification enzymes, the GSTM1 and GSTT1 null polymorphisms were extensively analyzed across the vast majority of the included studies, alongside GSTP1 and GSTM3 variants.

### Main results

Table 5 presents the genotypic frequencies and the calculated risk estimations (Odds Ratios) for the evaluated polymorphisms. The GSTM1 null genotype was consistently associated with an increased susceptibility to oral cancer in multiple studies. For instance, Katiyar et al. [13] and Buch et al. [11] reported significant Odds Ratios (OR = 1.87 and OR = 3.2, respectively) for the GSTM1 null genotype.

**Table 5:** Genotype frequencies, risk estimates, and statistical significance across the included studies

Author and year	Genotype frequencies in cases	Genotype frequencies in controls	Odds ratio (OR)	95% Confidence interval (CI 95%)	p value
ThekkePurakkal et al. [25]	GSTP1 105Val: Carriers (AG/GG) = 184 (47%); Non carriers = 203 (53%)	GSTP1 105Val: Carriers (AG/GG) = 234 (55%); Non carriers = 194 (45%)	0.71 (Protective for GSTP1 105Val carriers)	0.53 to 0.95	< 0.05 (Significant)
Maurya et al. [17]	CYP1A1*2A (Oral Cancer): Wt = 173; Het = 97; Mut = 30	CYP1A1*2A (Total): Wt = 451; Het = 254; Mut = 44	1.77 (For oral cancer with CYP1A1*2A mutation)	1.08 to 2.91	< 0.05 (Significant)
Zhuo et al. [21]	Total Meta analysis = 1515	Total Meta analysis = 2233	1.74 (Only for Asian subgroup Val/Val vs Ile/Ile)	1.04 to 2.90	0.033
Balaji et al. [19]	CYP2E1 (rs3813867): c1/c1 = 151; c1/c2 = 6; c2/c2 = 0	CYP2E1 (rs3813867): c1/c1 = 125; c1/c2 = 7; c2/c2 = 0	0.710 (For dominant mutant vs wild type)	0.232 to 2.166	> 0.05 (Not significant)
Katoh et al. [12]	GSTM1 null: 54 (58.7%)	GSTM1 null: 68 (46.3%)	1.81 (For GSTM1 null)	1.00 to 3.28	< 0.05 (Significant for GSTM1)
Hung et al. [9]	CYP2E1 (c1/c2 + c2/c2): 21 (51.2%)	CYP2E1 (c1/c2 + c2/c2): 46 (37.4%)	4.7 (Only in non betel chewers subgroup)	1.1 to 20.2	< 0.05 (Significant gene environment interaction)
Bartake et al. [26]	L432V (OSCC): CC=0; CG=14; GG=8. (OED): CC=1; CG=12; GG=9	L432V (Tobacco Controls): CC=1; CG=5; GG=16	5.95 (For OSCC with CG genotype vs Exposed controls)	N/A	< 0.05 (Significant for increased risk in CG)
Singh et al. [27]	N/A (MDR approach)	N/A	N/A (Best 3 locus model: Tobacco + CYP1A1*2C + p53 Intron 3/Intron 6)	N/A	0.01 (For the MDR model)
Katiyar et al. [13]	GSTM1 null: 569 (45.52%); GSTT1 null: 317 (25.36%)	GSTM1 null: 385 (30.80%); GSTT1 null: 245 (19.60%)	1.87 (GSTM1 null); 1.39 (GSTT1 null)	1.59 to 2.21 (GSTM1); 1.15 to 1.68 (GSTT1)	< 0.05 (Significant for both null genotypes)
Li et al. [22]	Total Meta analysis = 1306	Total Meta analysis = 1484	2.715 (For GSTM1 null vs present, only in Mainland China)	2.17 to 3.38	Significant
Rajesh et al. [16]	c.341C>T: CC=67; CT=18; TT=5	c.341C>T: CC=167; CT=12; TT=1	4.4 (For CT+TT vs CC dominant model)	2.1 to 9.2	0.00006 (Significant)
Krüger et al. [20]	GSTM1 null: 57 (57%); GSTT1 null: 22 (22%)	GSTM1 null: 53 (57%); GSTT1 null: 17 (18%)	1.00 (GSTM1 null); 1.26 (GSTT1 null)	0.57 to 1.77 (GSTM1); 0.62 to 2.56 (GSTT1)	> 0.05 (Not significant overall)
Singh et al. [28]	GSTM1 null: 53 (43.4%); GSTT1 null: 28 (23.0%)	GSTM1 null: 42 (33.1%); GSTT1 null: 17 (13.4%)	1.55 (GSTM1 null); 1.92 (GSTT1 null)	0.92 to 2.60 (GSTM1); 0.99 to 3.73 (GSTT1)	0.05 (For GSTT1)
Koch et al. [29]	GSTM1 null: 15 (54%); GSTM1 positive: 13 (46%)	N/A (No healthy control group)	N/A	N/A	N/A (Study showed GSTM1 null is associated with larger T3/T4 tumors)
Chen et al. [30]	GSTP1 (A/G + G/G): 63 (38.41%); GSTA1 (C/T + T/T): 44 (26.83%)	GSTP1 (A/G + G/G): 82 (29.93%); GSTA1 (C/T + T/T): 60 (21.90%)	1.53 (AOR for GSTP1 variant G allele); 1.33 (AOR for GSTA1 variant T allele)	1.01 to 2.31 (GSTP1); 0.84 to 2.13 (GSTA1)	Significant for GSTP1 (< 0.05)
Bathi et al. [31]	Oral Cancer GSTM1 null: 16 (53.3%); GSTT1 null: 20 (66.6%)	GSTM1 null: 36 (60%); GSTT1 null: 45 (75%)	1.31 (RR for GSTM1 null); 1.50 (RR for GSTT1 null)	0.465 to 2.737 (GSTM1); 0.457 to 3.108 (GSTT1)	< 0.05 (Authors reported statistical significance)
Chatterjee et al. [32]	Leukoplakia GSTM1 null: 32.69%; GSTT1 null: 11.50%	GSTM1 null: 20.3%; GSTT1 null: 6.25%	0.83 (GSTM1 null); 1.94 (GSTT1 null)	1.48 to 5.42 (GSTM1); 2.61 to 18.54 (GSTT1)	> 0.05 (Not significant for GSTM1); < 0.05 (Significant for GSTT1)
Datta et al. [15]	mt A12308G (A allele): 88%	mt A12308G (A allele): 82%	1.7 (For major A allele in overall cancer)	1.1 to 2.6	0.02 (Significant)
Komiya et al. [18]	GSTA1 (*A/*B + *B/*B): 33.3% in male smokers	GSTA1 (*A/*B + *B/*B): 19.6% in male smokers	1.97 (For variant genotypes in male smokers)	1.02 to 3.79	< 0.05 (Significant interaction with smoking)
Sikdar et al. [14]	GSTP1 105 (ile/ile): 149 (58%)	GSTP1 105 (ile/ile): 128 (49%)	2.0 (For ile/ile genotype at codon 105)	1.0 to 2.0	0.04 (Significant)
Buch et al. [11]	GSTM1 null: 146 (49.2%)	GSTM1 null: 111 (24.0%)	3.2 (For GSTM1 null)	2.4 to 4.3	< 0.001 (Significant)

OR: Odds Ratio; CI: Confidence Interval; p value: Probability value; Wt: Wild type; Het: Heterozygous; Mut: Mutant; RR: Relative Risk; AOR: Adjusted Odds Ratio; MDR: Multifactor Dimensionality Reduction; N/A: Not applicable or not available. Statistical significance was set at p < 0.05

Conversely, the results for the CYP1A1 and GSTT1 polymorphisms showed more variability. While some studies, such as Maurya et al. [17], found a significant association between CYP1A1*2A mutation and oral cancer risk (OR = 1.77), the broader literature sometimes indicates no obvious association depending on the specific variant and population. Interestingly, the analysis revealed crucial gene-environment interactions. While some studies merely reported stratified associations, others conducted formal statistical interaction testing. Hung et al. [9] highlighted an increased risk for CYP2E1 variants specifically in the non-betel chewers subgroup, and Komiya et al. [18] demonstrated a significant risk increase for GSTA1 variants exclusively among male smokers.

Although many studies reported increased risks, the literature also presents contradictory and null findings that highlight the complexity of these genetic interactions. Notably, ThekkePurakkal et al. reported a protective association for GSTP1 105Val carriers (OR = 0.71, 95% CI 0.53 to 0.95) among Canadian Caucasians [25]. Similarly, Balaji et al. found no significant association between CYP2E1 polymorphisms and oral cancer susceptibility in South Indians [19]. On the other hand, Zhuo et al. demonstrated through a meta-analysis that the CYP1A1 Ile462Val polymorphism significantly increases oral carcinoma risk in Asian populations [21]. Furthermore, Bartake et al. [26] found a significant risk increase for the CYP1B1 L432V variant in oral squamous cell carcinoma, and Datta et al. [15] highlighted the role of mitochondrial loci, reporting an increased risk associated with the mt A12308G locus.

## Discussion

Oral carcinogenesis is a complex biological and molecular process arising from the progressive disruption of cellular homeostasis in response to environmental carcinogens and the individual’s capacity to metabolize, detoxify, and repair the resulting molecular damage. The evidence compiled in this review suggests that polymorphisms in genes encoding Phase I and Phase II xenobiotic-metabolizing enzymes do not act independently. Rather, their potential effects may depend substantially on the type, intensity, and duration of exposure to carcinogens derived from tobacco, alcohol, and areca nut or betel quid [8]. Thus, genetic variation in xenobiotic metabolism may contribute to inter-individual differences in susceptibility to DNA damage and oral carcinogenesis.

From a mechanistic perspective, the interplay between Phase I bioactivation, Phase II detoxification, and environmental exposure is particularly relevant for understanding individual susceptibility. Experimental and epidemiological evidence indicates that polymorphisms in CYP1A1 and CYP1B1 may modify the metabolic activation of tobacco-derived polycyclic aromatic hydrocarbons, whereas CYP2E1 variants may influence pathways involved in ethanol metabolism and the bioactivation of certain nitrosamines derived from tobacco and betel quid [9, 10]. Conversely, individuals carrying GSTM1 or GSTT1 null genotypes may have reduced capacity for the conjugation and detoxification of specific electrophilic metabolites. Similarly, functional variation in GSTP1 and GSTM3 may alter the efficiency of Phase II detoxification [5, 15]. The resulting imbalance between metabolic activation and detoxification may favor the persistence of reactive intermediates, oxidative stress, DNA adduct formation, and genomic instability. Collectively, these observations support the hypothesis that susceptibility to oral cancer and oral potentially malignant disorders (OPMDs) may be better explained by combinations of metabolic genotypes and sustained exposure to established carcinogens rather than by isolated polymorphisms alone [33].

Among Phase II enzymes, the glutathione S-transferase (GST) family represents an important component of cellular xenobiotic detoxification. GST enzymes catalyze the conjugation of reduced glutathione with a broad range of electrophilic compounds, generally facilitating their subsequent metabolism and elimination. GSTM1 and GSTT1 are particularly relevant because common gene deletions can result in null genotypes and consequently in the absence of functional enzyme activity from the corresponding locus [5]. The prevalence of these null genotypes varies considerably among populations, making genetic ancestry and population structure important considerations when interpreting association studies.

The potential consequences of GSTM1 and GSTT1 deficiency may become more relevant in the context of sustained exposure to tobacco smoke or areca nut/betel quid. Areca nut contains several biologically active compounds, including arecoline, which can contribute to oxidative stress and other cellular alterations. Tobacco smoke, meanwhile, contains numerous carcinogenic and genotoxic constituents, including tobacco-specific nitrosamines and polycyclic aromatic hydrocarbons. Reduced GST-mediated detoxification may therefore increase the persistence of electrophilic metabolites and potentially facilitate oxidative damage, lipid peroxidation, and DNA adduct formation. These molecular alterations may contribute to genomic instability and disruption of pathways regulating cell proliferation, DNA repair, and apoptosis. Consistent with this mechanistic framework, studies such as those by Katiyar et al. [13] and Li et al. [22] have reported associations between GST polymorphisms and oral cancer susceptibility, while Lou et al. [33] reported associations of GSTM1 and GSTT1 null genotypes with OPMDs, particularly oral leukoplakia and oral submucous fibrosis. Nevertheless, the magnitude and consistency of these associations vary across populations, emphasizing the need to consider environmental exposure and genetic background when interpreting the available evidence.

In contrast to Phase II detoxification, Phase I enzymes, particularly members of the cytochrome P450 (CYP) superfamily, can generate reactive metabolites during the oxidative metabolism of xenobiotics. Although this metabolic transformation generally facilitates subsequent detoxification and elimination, some substrates can undergo bioactivation into chemically reactive intermediates capable of interacting with cellular macromolecules, including DNA [21]. Consequently, Phase I activity may have either detoxifying or bioactivating consequences depending on the substrate, enzyme, genetic variant, and cellular context.

Tobacco smoke represents a major source of chemical carcinogens relevant to oral carcinogenesis and contains polycyclic aromatic hydrocarbons that require metabolic activation to exert their genotoxic effects. Genetic variants in CYP1A1 and CYP1B1 may alter the activity or expression of these enzymes and consequently modify the formation of reactive metabolites. For example, the CYP1A1*2A variant reported by Maurya et al. [17] and the L432V variant described by Bartake et al. [26] have been investigated in relation to susceptibility to tobacco-related carcinogenesis. Increased or altered CYP-mediated bioactivation may enhance the formation of reactive intermediates capable of generating DNA adducts and other forms of molecular damage. If such damage exceeds the capacity of cellular repair and protective mechanisms, it may contribute to persistent genomic alterations, epithelial dysplasia, and ultimately malignant transformation.

Taken together, these findings support a model in which oral carcinogenesis results from the cumulative interaction between carcinogen exposure, Phase I metabolic activation, Phase II detoxification capacity, oxidative stress, DNA repair, and individual genetic background. Importantly, the presence of a single susceptibility polymorphism should not be interpreted as sufficient to determine cancer development. Rather, the available evidence suggests that genetic variants may modify the biological consequences of environmental exposure, with the overall risk emerging from the interaction of multiple genetic and environmental determinants.

The interaction between genetic susceptibility and alcohol exposure may further contribute to oral carcinogenesis, particularly through pathways involving the cytochrome P450 2E1 (CYP2E1) enzyme. Chronic ethanol consumption can induce CYP2E1 expression and activity, potentially increasing the formation of acetaldehyde, a recognized carcinogenic and mutagenic metabolite capable of generating DNA adducts and cross-links. Genetic variation in CYP2E1 may further modulate individual differences in xenobiotic metabolism; however, the extent to which specific CYP2E1 polymorphisms influence acetaldehyde-mediated genotoxicity in oral tissues remains to be fully established.

CYP2E1 is also involved in the metabolic activation of several tobacco- and betel quid-derived compounds, including nitrosamines, thereby potentially contributing to the generation of reactive metabolites and oxidative stress [9]. In addition to polycyclic aromatic hydrocarbons and nitrosamines, tobacco smoke contains carcinogenic aromatic amines and heterocyclic amines, including o-toluidine, 2-naphthylamine, and 4-aminobiphenyl [6]. These compounds undergo metabolism through human arylamine N-acetyltransferases (NATs), particularly NAT1 and NAT2 [34]. Functional polymorphisms in NAT1 and NAT2 can result in differences in acetylator phenotype and may consequently influence the balance between metabolic activation and detoxification of aromatic amine carcinogens [35]. Therefore, characterization of NAT1 and NAT2 variants provides an additional dimension for understanding interindividual differences in susceptibility to oral squamous cell carcinoma and highlights the importance of xenobiotic-metabolizing pathways in the complex interplay between genetic background and environmental exposure [36].

The potential impact of these interactions becomes particularly relevant when multiple susceptibility factors coexist. Individuals carrying genetic variants associated with increased Phase I bioactivation and reduced Phase II detoxification, together with sustained exposure to tobacco, alcohol, or betel quid, may experience a greater cumulative burden of reactive metabolites and oxidative stress. This altered metabolic balance could contribute to DNA damage, impaired genomic stability, and, ultimately, an increased susceptibility to oral squamous cell carcinoma. Similarly, the combined influence of genetic susceptibility and carcinogenic exposure may be relevant to the development of oral potentially malignant disorders (OPMDs), which represent clinically identifiable conditions associated with an increased risk of malignant transformation [33]. Nevertheless, the available evidence should be interpreted cautiously, as the magnitude and consistency of these gene–environment interactions remain heterogeneous across populations and study designs.

A critical appraisal of this literature must also consider important epidemiological limitations [23]. Although major environmental confounders, including tobacco use, alcohol consumption, and betel quid exposure, are frequently controlled for or matched between cases and controls, as summarized in Table 3, several potentially relevant variables are inconsistently assessed or reported. Population stratification represents a particular concern in genetic association studies because differences in ancestry between cases and controls may generate spurious associations. Similarly, deviations from Hardy–Weinberg equilibrium (HWE) among control groups may reflect methodological problems, population structure, genotyping errors, or, in some circumstances, true biological characteristics of the population and therefore warrant careful evaluation. In addition, the potential contribution of human papillomavirus (HPV) infection to oral carcinogenesis [3] remains insufficiently addressed in several studies and may represent an additional source of heterogeneity or confounding. Collectively, these limitations may affect the reproducibility and interpretation of reported genetic associations and reinforce the need for adequately powered, multicenter prospective studies incorporating standardized exposure assessment, genetic ancestry, HPV status, and comprehensive adjustment for established environmental risk factors [23].

Finally, the consolidated evidence suggests that the development of oral cancer extends beyond classical metabolic pathways and may involve a broader and systemic impairment of genomic and cellular protection mechanisms. The statistical and biological interactions reported by Singh et al. [27], linking metabolic polymorphisms to alterations in the p53 tumor suppressor pathway, together with the mitochondrial instability associated with the mtDNA A12308G locus described by Datta et al. [15], support the concept that cumulative genomic and mitochondrial damage may progressively compromise cellular homeostasis. Such alterations may enhance endogenous oxidative stress, impair mitochondrial function, and interfere with DNA repair and apoptotic responses, thereby favoring the persistence and accumulation of molecular lesions associated with malignant transformation [37]. Collectively, these findings suggest that the progressive loss of genomic integrity and cellular repair capacity may represent a critical transition toward a state of increased susceptibility to oral carcinogenesis.

## Concluding remarks

This review concludes that susceptibility to oral cancer is closely modulated by genetic polymorphisms in xenobiotics metabolizing enzymes. The deficiency or total absence of Phase II detoxification enzymes, particularly represented by the GSTM1 null and GSTT1 null genotypes, acts in synergy with the hyperactivity of Phase I enzymes, such as the CYP family variants. This combination establishes an environment of extreme cellular and genotoxic vulnerability in the mucosa of the oral cavity.

Furthermore, the available evidence suggests that this intrinsic genetic susceptibility may be substantially influenced by environmental exposures, particularly chronic consumption of tobacco, alcohol, and areca nut or betel quid. These interactions may contribute to the accumulation of genomic damage and increase the likelihood of malignant transformation in genetically susceptible individuals. From a clinical perspective, the identification of specific polymorphic profiles represents a promising avenue for the development of predictive biomarkers; however, their clinical utility requires validation in well-designed prospective studies and diverse populations. If subsequently validated, such biomarkers could potentially contribute to individualized risk stratification and support the implementation of targeted screening and preventive strategies, particularly among individuals with sustained exposure to established oral carcinogens.

## Supplementary Information

Below is the link to the electronic supplementary material.

Supplementary file1 (DOCX 2257 KB)

## Data Availability

No datasets were generated or analysed during the current study.
