# Supplementary material for: The impact of genetic polymorphisms in xenobiotic-metabolizing enzymes and the risk of oral cancer: a critical review
Source: Med Oncol. 2026 Oct 8;43(11):312. doi: 10.1007/s12032-026-03428-x (PMC13649812; doi:10.1007/s12032-026-03428-x)
Supplement: Supplementary file 1 [file 12032_2026_3428_MOESM1_ESM.docx]

**Table 1 - Search strategy**

| **Electronic databases used** | **Search strategy (April, 2026)** |
| --- | --- |
| PubMed https://www.ncbi.nlm.nih.gov/pubmed/  Scopus https://www.scopus.com  Web of Science  https://www.webofscience.com/wos/alldb/ basic-search | (“Oral cancer” OR “Oral squamous cell carcinoma” OR OSCC OR “Oral potentially malignant disorders” OR Leukoplakia OR “Oral submucous fibrosis”) AND (Polymorphism OR “Single nucleotide polymorphism” OR SNP OR Genotype) AND (“Cytochrome P450” OR CYP1A1 OR CYP2E1 OR “Glutathione S-transferase” OR GSTM1 OR GSTT1 OR GSTP1 OR “Metabolizing enzymes”) |

**Table 2 - Main demographic and methodological characteristics of the included studies.**

| **Author and Year** | **Country** | **Ethnicity** | **Study Design** | **Clinical Condition** | **Sample Size (Cases/Controls)** |
| --- | --- | --- | --- | --- | --- |
| ThekkePurakkal et al., 2019 | Canada | Caucasians | Case-control | HNSCC | 389 / 429 |
| Maurya et al., 2015 | India | Indo-European | Case-control | HNSCC | 750 / 750 |
| Zhuo et al., 2012 | China | Multiple | Meta-analysis | OSCC | 1515 / 2233 |
| Balaji et al., 2011 | India | South Asian | Case-control | OSCC | 157 / 132 |
| Katoh et al., 1999 | Japan | Japanese | Case-control | OSCC | 92 / 147 |
| Hung et al., 1997 | Taiwan | Asian | Case-control | OSCC | 41 / 123 |
| Bartake et al., 2024 | India | South Asian | Case-control | OED and OSCC | 44 / 44 |
| Singh et al., 2023 | India | Indian (Gujarat) | Case-control | OSCC | 121 / 121 |
| Katiyar et al., 2020 | India | Indo-European | Case-control | HNSCC | 1250 / 1250 |
| Li et al., 2018 | China | Chinese | Meta-analysis | OSCC | 1306 / 1484 |
| Rajesh et al., 2018 | India | Indian | Case-control | OSCC | 90 / 180 |
| Krüger et al., 2015 | Germany | Caucasian | Case-control | OSCC | 100 / 93 |
| Singh et al., 2014 | India | Indian (Gujarati) | Case-control | OSCC | 122 / 127 |
| Koch et al., 2010 | Germany | Caucasian | Case-series | OSCC | 28 / 0 |
| Chen et al., 2010 | Taiwan | Taiwanese | Case-control | OSCC | 164 / 274 |
| Bathi et al., 2009 | India | Indian | Case-control | Oral cancer, Leukoplakia, OSF | 90 / 60 |
| Chatterjee et al., 2009 | India | Indian (Tribal/Nontribal) | Case-control | Leukoplakia | 52 / 217 |
| Datta et al., 2007 | India | Indian (Bengalee) | Case-control | OSCC and Leukoplakia | 310 / 389 |
| Komiya et al., 2005 | Japan | Japanese | Case-control | OSCC | 97 / 457 |
| Sikdar et al., 2004 | India | Indian (Bengalee) | Case-control | OSCC and Leukoplakia | 256 / 259 |
| Buch et al., 2002 | India | Indian | Case-control | OSCC | 297 / 450 |

HNSCC = Head and neck squamous cell carcinoma; OSCC = Oral squamous cell carcinoma, OED = Oral epithelial dysplasia; OSF = Oral submucous fibrosis.

**Table 3 – Risk Factors and Habits**

| **Author and year** | **Age (Cases/ Controls)** | **Gender (Cases/ Control)** | **Tobacco Smoking** | **Alcohol consumption** | **Betel quid/ smokeless tabacco** |
| --- | --- | --- | --- | --- | --- |
| ThekkePurakkal et al., 2019 | 61 / 61 years | 291M, 98F / 298M, 131F | Yes (Evaluated in pack years: nonsmokers, moderate, heavy) | Yes (Ethanol frequency evaluated in ml/day) | N/A |
| Maurya et al., 2015 | 53 ± 9 / 54 ± 6 years | 750M, 0F / 750M, 0F (Only male sample) | Yes (67% cases / 29% controls) | Yes (42% cases / 31% controls) | Yes (Tobacco chewing: 53% cases / 32% controls) |
| Zhuo et al., 2012 | N/A | N/A | N/A (Meta analysis did not provide isolated statistics) | N/A | N/A |
| Balaji et al., 2011 | 55.1 / 53.1 years | 54.8% M / 34.8% M | Evaluated and matched | Evaluated and matched | N/A |
| Katoh et al., 1999 | 62.4 / 69.9 years | 60.9% M / 61.9% M | Yes (63% cases / 34% controls) | Yes (56.7% cases / 53.1% controls) | N/A |
| Hung et al., 1997 | 54.1 / 51.7 years | Only male sample (100% M) | Yes (90.2% cases / 65.8% controls) | Yes (65.9% cases / 40.7% controls) | Yes (73.2% cases / 12.2% controls) |
| Bartake et al., 2024 | 54.6 (OSCC) and 49.6 (OED) / 43.2 (CWT) and 37.6 (CWOT) | OSCC (72.7% M) and OED (86.4% M) / CWT (63.6% M) and CWOT (36.4% M) | Yes (All cases and 50% of controls used tobacco) | Not evaluated as grouping variable | N/A |
| Singh et al., 2023 | 42.6 / 40.2 years | 81.2% M / 92.3% M | Yes (Included in general tobacco use: 85.1% cases / 47.9% controls) | Yes (Considered as risk factor, but frequency not detailed) | Yes (Mentioned gutkha and pan masala) |
| Katiyar et al., 2020 | 44.47 / 51.55 years | 100% M / 100% M | Yes (Smokers: 67.6% cases / 29.3% controls) | Yes (35.1% cases / 25.9% controls) | Yes (Tobacco chewers: 52% cases / 32% controls) |
| Li et al., 2018 | N/A | N/A | N/A | N/A | N/A |
| Rajesh et al., 2018 | 54.2 ± 12.03 / 53.7 ± 12.5 years | 20M, 70F / 43M, 137F | Yes (Evaluated as overall tobacco consumption) | Yes (Only 5 cases reported alcohol use) | Yes (100% of cases used betel quid or chewable tobacco) |
| Krüger et al., 2015 | Not informed | Not informed | Yes (Smokers vs Nonsmokers analyzed) | Not evaluated as primary variable | N/A |
| Singh et al., 2014 | 44.4 ± 1.0 / 32.2 ± 0.72 years | 101M, 21F / 116M, 11F | Yes (85.2% cases / 48.8% controls) | Evaluated as mixed habits (17.2% of cases) | Yes (Exclusive chewers: 54.9% cases / 41.7% controls) |
| Koch et al., 2010 | 67 / Not informed years | 21M, 7F / N/A | Yes (Mentioned as general risk factor, no frequencies provided) | Yes (Mentioned as general risk factor) | N/A |
| Chen et al., 2010 | 53.35 ± 11.94 / 55.87 ± 17.50 years | Matched (frequencies not detailed in text) | Yes (Adjusted as confounding factor) | Yes (Adjusted as confounding factor) | Yes (Adjusted as confounding factor) |
| Bathi et al., 2009 | 21 to 75 years range | Matched (frequencies not detailed) | Yes (Control group divided into 30 smokers and 30 nonsmokers) | N/A | Yes (Cases had smoking, chewing or mixed habits) |
| Chatterjee et al., 2009 | Mean 28.88 years for target population | Overall population: 68M, 149F | Yes (Evaluated by shots/day) | N/A | Yes (Mentioned raw tobacco and betel nut usage) |
| Datta et al., 2007 | 55 ± 11 / 49 ± 12 years | 198M, 112F / 302M, 87F (For Cancer vs Control) | Yes (17% Cases / 37% Controls) | Evaluated but excluded due to low frequency | Yes (57% Cases / 44% Controls) |
| Komiya et al., 2005 | 62.8 / 67.2 years | 62M, 35F / 284M, 173F | Yes (Adjusted as confounding factor) | Not collected for controls | N/A |
| Sikdar et al., 2004 | 55 ± 11.6 / 53 ± 11.0 years | 168M, 88F / 183M, 76F (For Cancer vs Control) | Yes (Evaluated as light/heavy pack years) | Excluded from analysis (low frequency) | Yes (Evaluated as light/heavy chewing years) |
| Buch et al., 2002 | 52.2 ± 0.69 / 42.9 ± 0.53 years | Not explicitly detailed in tables | Yes (Bidi and cigarette) | N/A | Yes (Chewing tobacco with or without betel quid) |

HNSCC: Head and neck squamous cell carcinoma; OSCC: Oral squamous cell carcinoma; OED: Oral epithelial dysplasia; OSF: Oral submucous fibrosis; M: Male; F: Female; N/A: Not applicable or not available.

**Table 4 – Molecular methodologies and specific genetic polymorphisms evaluated**

| **Author and Year** | **Source of DNA** | **Genotyping Method** | **Evaluated Genes** | **Polymorphism / SNP** |
| --- | --- | --- | --- | --- |
| ThekkePurakkal et al., 2019 | Oral exfoliated cells (buccal mucosa) | Real time PCR (TaqMan) | CYP1A1, CYP2A6, CYP2E1, GSTP1, CYP2D6, GSTM1 | CYP1A1*2A, CYP1A1*2C, CYP2A6*2, CYP2E1c2, GSTP1 105Val, null CYP2D6, GSTM1 null |
| Maurya et al., 2015 | Whole blood | Multiplex PCR (GSTM1) and PCR RFLP (CYPs) | CYP1A1, CYP1B1, CYP2E1, GSTM1 | CYP1A1*2A (Msp1), CYP1A1*2C (Ile/Val), CYP1B1*2, CYP1B1*3, CYP2E1*5B (RsaI), CYP2E1*6 (DraI), GSTM1 (null) |
| Zhuo et al., 2012 | N/A | Various (PCR RFLP, AS PCR, PCR SSCP in primary studies) | CYP1A1 | Ile462Val (exon7) / rs1048943 |
| Balaji et al., 2011 | Peripheral blood | TaqMan Fast Real Time PCR | CYP2E1 | rs6413419 (V1791), rs3813867 (PstI), rs2031920 (RsaI) |
| Katoh et al., 1999 | Peripheral leukocytes | Multiplex PCR (GSTs) and PCR RFLP (CYPs) | CYP1A1, CYP2E1, GSTM1, GSTT1, ALDH2 | CYP1A1 (Ile462Val), CYP2E1 (RsaI c1/c2), GSTM1 (null), GSTT1 (null) |
| Hung et al., 1997 | Peripheral white blood cells (WBCs) | PCR RFLP (CYP2E1) and PCR (GSTs) | CYP2E1, GSTM1, GSTT1 | CYP2E1 (RsaI c1/c2), GSTM1 (null), GSTT1 (null) |
| Bartake et al., 2024 | Whole blood (EDTA) | TaqMan RT PCR | CYP1B1 | Leu432Val / L432V (rs1056836) |
| Singh et al., 2023 | Peripheral blood lymphocytes | PCR RFLP and PCR SSCP | CYP1A1, CYP2E1, GSTM1, GSTT1, p53, MDM2 | CYP1A1*2A (rs1048943), CYP1A1*2C (rs4646903), CYP2E1*5B, CYP2E1*6 (rs6413432), GSTM1 (null), GSTT1 (null), p53 (rs17878362, rs1042522, rs1625895), MDM2 (rs2279744) |
| Katiyar et al., 2020 | Whole blood | Multiplex PCR (GSTM1, GSTT1) and PCR RFLP (GSTP1) | GSTM1, GSTT1, GSTP1 | GSTM1 (null), GSTT1 (null), GSTP1 (Ile105Val) |
| Li et al., 2018 | N/A | Meta analysis | GSTM1, GSTT1 | Null genotypes |
| Rajesh et al., 2018 | Tumor specimens (Cases) and Peripheral blood (Controls) | PCR RFLP | GSTP1 | c.341C>T (rs1138272) |
| Krüger et al., 2015 | Lymphocytes from venous blood (Cases) and Brush biopsy (Controls) | Real time PCR (TaqMan) | GSTM1, GSTT1 | Null genotypes (Deletion) |
| Singh et al., 2014 | Peripheral lymphocytes | PCR RFLP (CYP1A1*2A), PCR SSCP (CYP1A1*2C), and Multiplex PCR (GSTs) | CYP1A1, GSTM1, GSTT1 | CYP1A1*2A (MspI), CYP1A1*2C (Ile/Val), GSTM1 (null), GSTT1 (null) |
| Koch et al., 2010 | Peripheral blood (for DNA) and Brush biopsy (for mRNA) | PCR (for DNA) and Real time RT qPCR (for mRNA) | GSTM1 | Null genotype (homozygous deletion) |
| Chen et al., 2010 | Whole blood | PCR RFLP | GSTP1, GSTA1, GSTT1, GSTM1 | GSTP1 (Ile105Val / A>G), GSTA1 (C>T at 69), GSTT1 (null), GSTM1 (null) |
| Bathi et al., 2009 | Peripheral blood leukocytes | PCR | GSTM1, GSTT1 | Null genotypes |
| Chatterjee et al., 2009 | Peripheral blood leukocytes | PCR RFLP (CYP1A1) and Multiplex PCR (GSTs) | CYP1A1, GSTM1, GSTT1 | CYP1A1 (MspI: m1/m2), GSTM1 (null), GSTT1 (null) |
| Datta et al., 2007 | Whole blood (and affected tissues) | PCR RFLP | Mitochondrial loci, GSTP1, GSTM1, GSTM3 | mt A12308G, A11467G, G10398A, C10400T; GSTs (various) |
| Komiya et al., 2005 | Peripheral leukocytes | PCR RFLP | GSTA1 | *A/*B (*A: 567T, 69C, 52G, *B: 567G, 69T, 52A) |
| Sikdar et al., 2004 | Whole blood | Multiplex PCR, PCR, and PCR RFLP | GSTM1, GSTT1, GSTM3, GSTP1 | GSTM1 (null), GSTT1 (null), GSTM3 (3bp deletion A/B), GSTP1 (Ile105Val, Ala114Val) |
| Buch et al., 2002 | White blood cells | PCR and PCR RFLP | GSTM1, GSTM3, GSTT1 | GSTM1 (null), GSTM3 (A/B), GSTT1 (null) |

PCR: Polymerase chain reaction; PCR RFLP: Polymerase chain reaction restriction fragment length polymorphism; PCR SSCP: Polymerase chain reaction single strand conformation polymorphism; AS PCR: Allele specific polymerase chain reaction; RT PCR: Real time polymerase chain reaction; CYP: Cytochrome P450; GST: Glutathione S transferase; ALDH2: Aldehyde dehydrogenase 2; MDM2: Mouse double minute 2 homolog; SNP: Single nucleotide polymorphism.

**Table 5 – Genotype frequencies, risk estimates, and statistical significance across the included studies**

| **Author and Year** | **Genotype Frequencies in Cases** | **Genotype Frequencies in Controls** | **Odds Ratio (OR)** | **95% Confidence Interval (IC 95%)** | **p value** |
| --- | --- | --- | --- | --- | --- |
| ThekkePurakkal et al., 2019 | GSTP1 105Val: Carriers (AG/GG) = 184 (47%); Non carriers = 203 (53%) | GSTP1 105Val: Carriers (AG/GG) = 234 (55%); Non carriers = 194 (45%) | 0.71 (Protective for GSTP1 105Val carriers) | 0.53 to 0.95 | < 0.05 (Significant) |
| Maurya et al., 2015 | CYP1A1*2A (Oral Cancer): Wt = 173; Het = 97; Mut = 30 | CYP1A1*2A (Total): Wt = 451; Het = 254; Mut = 44 | 1.77 (For oral cancer with CYP1A1*2A mutation) | 1.08 to 2.91 | < 0.05 (Significant) |
| Zhuo et al., 2012 | Total Meta analysis = 1515 | Total Meta analysis = 2233 | 1.74 (Only for Asian subgroup Val/Val vs Ile/Ile) | 1.04 to 2.90 | 0.033 |
| Balaji et al., 2011 | CYP2E1 (rs3813867): c1/c1 = 151; c1/c2 = 6; c2/c2 = 0 | CYP2E1 (rs3813867): c1/c1 = 125; c1/c2 = 7; c2/c2 = 0 | 0.710 (For dominant mutant vs wild type) | 0.232 to 2.166 | > 0.05 (Not significant) |
| Katoh et al., 1999 | GSTM1 null: 54 (58.7%) | GSTM1 null: 68 (46.3%) | 1.81 (For GSTM1 null) | 1.00 to 3.28 | < 0.05 (Significant for GSTM1) |
| Hung et al., 1997 | CYP2E1 (c1/c2 + c2/c2): 21 (51.2%) | CYP2E1 (c1/c2 + c2/c2): 46 (37.4%) | 4.7 (Only in non betel chewers subgroup) | 1.1 to 20.2 | < 0.05 (Significant gene environment interaction) |
| Bartake et al., 2024 | L432V (OSCC): CC=0; CG=14; GG=8. (OED): CC=1; CG=12; GG=9. | L432V (Tobacco Controls): CC=1; CG=5; GG=16 | 5.95 (For OSCC with CG genotype vs Exposed controls) | N/A | < 0.05 (Significant for increased risk in CG) |
| Singh et al., 2023 | N/A (MDR approach) | N/A | N/A (Best 3 locus model: Tobacco + CYP1A1*2C + p53 Intron 3/Intron 6) | N/A | 0.01 (For the MDR model) |
| Katiyar et al., 2020 | GSTM1 null: 569 (45.52%); GSTT1 null: 317 (25.36%) | GSTM1 null: 385 (30.80%); GSTT1 null: 245 (19.60%) | 1.87 (GSTM1 null); 1.39 (GSTT1 null) | 1.59 to 2.21 (GSTM1); 1.15 to 1.68 (GSTT1) | < 0.05 (Significant for both null genotypes) |
| Li et al., 2018 | Total Meta analysis = 1306 | Total Meta analysis = 1484 | 2.715 (For GSTM1 null vs present, only in Mainland China) | 2.17 to 3.38 | Significant |
| Rajesh et al., 2018 | c.341C>T: CC=67; CT=18; TT=5 | c.341C>T: CC=167; CT=12; TT=1 | 4.4 (For CT+TT vs CC dominant model) | 2.1 to 9.2 | 0.00006 (Significant) |
| Krüger et al., 2015 | GSTM1 null: 57 (57%); GSTT1 null: 22 (22%) | GSTM1 null: 53 (57%); GSTT1 null: 17 (18%) | 1.00 (GSTM1 null); 1.26 (GSTT1 null) | 0.57 to 1.77 (GSTM1); 0.62 to 2.56 (GSTT1) | > 0.05 (Not significant overall) |
| Singh et al., 2014 | GSTM1 null: 53 (43.4%); GSTT1 null: 28 (23.0%) | GSTM1 null: 42 (33.1%); GSTT1 null: 17 (13.4%) | 1.55 (GSTM1 null); 1.92 (GSTT1 null) | 0.92 to 2.60 (GSTM1); 0.99 to 3.73 (GSTT1) | 0.05 (For GSTT1) |
| Koch et al., 2010 | GSTM1 null: 15 (54%); GSTM1 positive: 13 (46%) | N/A (No healthy control group) | N/A | N/A | N/A (Study showed GSTM1 null is associated with larger T3/T4 tumors) |
| Chen et al., 2010 | GSTP1 (A/G + G/G): 63 (38.41%); GSTA1 (C/T + T/T): 44 (26.83%) | GSTP1 (A/G + G/G): 82 (29.93%); GSTA1 (C/T + T/T): 60 (21.90%) | 1.53* (AOR for GSTP1 variant G allele); 1.33 (AOR for GSTA1 variant T allele) | 1.01 to 2.31 (GSTP1); 0.84 to 2.13 (GSTA1) | Significant for GSTP1 (< 0.05) |
| Bathi et al., 2009 | Oral Cancer GSTM1 null: 16 (53.3%); GSTT1 null: 20 (66.6%) | GSTM1 null: 36 (60%); GSTT1 null: 45 (75%) | 1.31 (RR for GSTM1 null); 1.50 (RR for GSTT1 null) | 0.465 to 2.737 (GSTM1); 0.457 to 3.108 (GSTT1) | < 0.05 (Authors reported statistical significance) |
| Chatterjee et al., 2009 | Leukoplakia GSTM1 null: 32.69%; GSTT1 null: 11.50% | GSTM1 null: 20.3%; GSTT1 null: 6.25% | 0.83 (GSTM1 null); 1.94 (GSTT1 null) | 1.48 to 5.42 (GSTM1); 2.61 to 18.54 (GSTT1) | > 0.05 (Not significant for GSTM1);  < 0.05 (Significant for GSTT1) |
| Datta et al., 2007 | mt A12308G (A allele): 88% | mt A12308G (A allele): 82% | 1.7 (For major A allele in overall cancer) | 1.1 to 2.6 | 0.02 (Significant) |
| Komiya et al., 2005 | GSTA1 (*A/*B + *B/*B): 33.3% in male smokers | GSTA1 (*A/*B + *B/*B): 19.6% in male smokers | 1.97 (For variant genotypes in male smokers) | 1.02 to 3.79 | < 0.05 (Significant interaction with smoking) |
| Sikdar et al., 2004 | GSTP1 105 (ile/ile): 149 (58%) | GSTP1 105 (ile/ile): 128 (49%) | 2.0 (For ile/ile genotype at codon 105) | 1.0 to 2.0 | 0.04 (Significant) |
| Buch et al., 2002 | GSTM1 null: 146 (49.2%) | GSTM1 null: 111 (24.0%) | 3.2 (For GSTM1 null) | 2.4 to 4.3 | < 0.001 (Significant) |

OR: Odds Ratio; CI: Confidence Interval; p value: Probability value; Wt: Wild type; Het: Heterozygous; Mut: Mutant; RR: Relative Risk; AOR: Adjusted Odds Ratio; MDR: Multifactor Dimensionality Reduction; N/A: Not applicable or not available. Statistical significance was set at p < 0.05. * Adjusted Odds Ratio (AOR) as reported by the primary study; unmarked values represent crude estimates.
